# Association between male circumcision and women’s biomedical health outcomes: a systematic review

**DOI:** 10.1016/S2214-109X(17)30369-8

**Published:** 2017-11

**Authors:** Jonathan M Grund, Tyler S Bryant, Inimfon Jackson, Kelly Curran, Naomi Bock, Carlos Toledo, Joanna Taliano, Sheng Zhou, Jorge Martin del Campo, Ling Yang, Apollo Kivumbi, Peizi Li, Sherri Pals, Stephanie M Davis

**Affiliations:** US Centers for Disease Control and Prevention, Division of Global HIV and TB, Atlanta, GA, USA (J M Grund MPH, N Bock MD, C Toledo PhD, S Pals PhD, S M Davis MD); Johns Hopkins Bloomberg School of Public Health, Baltimore, MD, USA (T S Bryant MHS, I Jackson MPH, S Zhou MBBS, J M del Campo MD, L Yang ScM, A Kivumbi MPH, P Li MD); Jhpiego, Baltimore, MD, USA (K Curran MPH); and US Centers for Disease Control and Prevention, Library Science Branch, Division of Public Health Information Dissemination, Center for Surveillance, Epidemiology, and Laboratory Services, Atlanta, GA, USA (J Taliano MLS)

## Abstract

**Background:**

Male circumcision reduces men’s risk of acquiring HIV and some sexually transmitted infections from heterosexual exposure, and is essential for HIV prevention in sub-Saharan Africa. Studies have also investigated associations between male circumcision and risk of acquisition of HIV and sexually transmitted infections in women. We aimed to review all evidence on associations between male circumcision and women’s health outcomes to benefit women’s health programmes.

**Methods:**

In this systematic review we searched for peer-reviewed and grey literature publications reporting associations between male circumcision and women’s health outcomes up to April 11, 2016. All biomedical (not psychological or social) outcomes in all study types were included. Searches were not restricted by year of publication, or to sub-Saharan Africa. Publications without primary data and not in English were excluded. We extracted data and assessed evidence on each outcome as high, medium, or low consistency on the basis of agreement between publications; outcomes found in fewer than three publications were indeterminate consistency.

**Findings:**

60 publications were included in our assessment. High-consistency evidence was found for five outcomes, with male circumcision protecting against cervical cancer, cervical dysplasia, herpes simplex virus type 2, chlamydia, and syphilis. Medium-consistency evidence was found for male circumcision protecting against human papillomavirus and low-risk human papillomavirus. Although the evidence shows a protective association with HIV, it was categorised as low consistency, because one trial showed an increased risk to female partners of HIV-infected men resuming sex early after male circumcision. Seven outcomes including HIV had low-consistency evidence and six were indeterminate.

**Interpretation:**

Scale-up of male circumcision in sub-Saharan Africa has public health implications for several outcomes in women. Evidence that female partners are at decreased risk of several diseases is highly consistent. Synergies between male circumcision and women’s health programmes should be explored.

**Funding:**

US Centers for Disease Control and Prevention and Jhpiego

## Introduction

Male circumcision has been shown to reduce the risk of HIV acquisition in men due to heterosexual exposure in three randomised controlled trials (RCTs).^1–3^ Shortly after the RCTs, WHO and UNAIDS recommended that voluntary medical male circumcision should be implemented as an intervention for HIV prevention.^4^ Voluntary medical male circumcision is now an essential component of the HIV prevention strategy in sub-Saharan Africa, with almost 15 million circumcisions done between 2007 and 2016.^5^ To maximise the effect of voluntary medical male circumcision on HIV incidence, UNAIDS has set a target to circumcise 90% of men aged 15–29 years in priority countries in eastern and southern Africa by 2021, totalling 27 million additional voluntary medical male circumcisions.^6^ Circumcision also provides men with other clinical benefits, including reduced incidence of herpes simplex virus type 2 (HSV-2) infection and prevalence of human papillomavirus (HPV) infection^7^ (including high-risk HPV subtypes),^8,9^ penile cancer,^10^ and genital ulcer disease.^3^ Meta-analyses have been done on these outcomes in men.^11,12^

WHO and UNAIDS noted in their recommendation that voluntary medical male circumcision programmes should promote improved health for women;^4^ evidence exists that male circumcision is associated with protection from some diseases in women. Protection could either be direct (ie, decreased infectiousness of men with HIV or sexually transmitted infections) or indirect (ie, decreased susceptibility of men to infection and therefore women’s exposure to infected partners). A 2009 meta-analysis by Weiss and colleagues^13^ found lower HIV prevalence in women in countries with high prevalences of circumcision than in countries with low prevalences. Moreover, secondary analyses of the circumcision RCTs supported the data from observational studies, showing that male circumcision protected female partners from other sexually transmitted infections, including bacterial vaginosis, trichomonas,^14^ and HPV.^9^

We aimed to consolidate existing data on the association of male circumcision with, and its effect on, biomedical health outcomes in women, and to clarify the implications of male circumcision on women’s health. Our findings are of greatest relevant to sub-Saharan Africa because of the regional scale-up of voluntary medical male circumcision, but are intended to be applicable globally.

## Methods

### Search strategy and selection criteria

For this systematic review we searched published and grey literature for publications reporting associations between male circumcision and biomedical (as opposed to psychological or social) health, or sexual satisfaction or function outcomes in women, as well as women’s knowledge of selected biomedical facts about circumcision. Because observational and interventional studies were included, “association” refers to findings from both study types, whereas “effect” refers to findings from interventional studies only. The search strategy was developed for use in MEDLINE (panel), and thereafter modified based on the syntax and capabilities of subsequent databases. Searches were not restricted by study design or year, or to sub-Saharan Africa because: some relevant outcomes might not have been studied in sub-Saharan Africa; the biological mechanisms underlying the associations of male circumcision with sexually transmitted infections are universal; and the potential relevance of findings to women’s health programmes is global despite its greatest relevance in sub-Saharan Africa, resulting from the regional scale-up of voluntary medical male circumcision. Any biomedical outcomes were included and results on women’s knowledge about voluntary medical male circumcision and sexual satisfaction and function will be reported separately.

**Figure 1:**
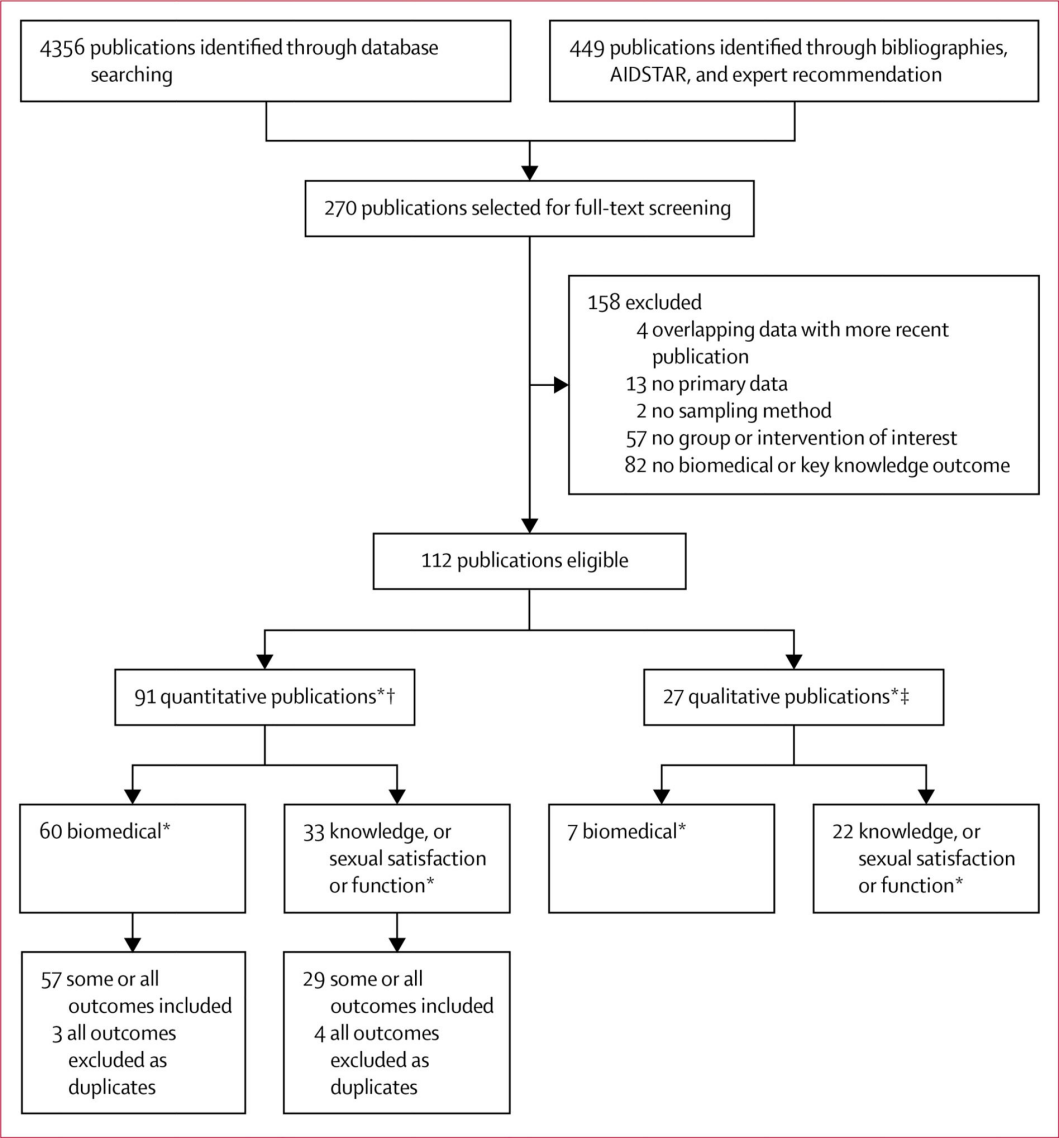
Publication selection flow diagram *Some publications provided biomedical and knowledge data, or qualitative and quantitative data, or all of these. The number of publications in these boxes are not a sum of the total publications in the parent box immediately above. †Articles reporting quantitative results, with or without qualitative results. ‡Articles reporting only qualitative results.

Databases of peer-reviewed literature included MEDLINE, EMBASE, Global Health, PsychInfo, CINAHL, Cochrane Library, Sociological Abstracts (Proquest), Scopus, and the African Index Medicus. Grey literature sources included OPENGREY, Greylit.org, National Technical Information Service, PsyExtra, and conference abstracts from international HIV conferences: the Conference on Retroviruses and Opportunistic Infections; International AIDS Society; and International Society for Sexually Transmitted Disease Research. These searches were last updated on April 11, 2016. Full reports of the National Demographic and Health Survey and AIDS Indicator Survey from 2008 onward, which contain questions about male circumcision, and the AIDSTAR^15^ resource website were browsed. In 2015, bibliographies of key review publications of others obtained through expert recommendations were also searched (figure 1). We use “publication” to refer to individual sources of data including journal publications, abstracts, white papers, and other sources, and “study” to refer to data collection protocols, which sometimes resulted in multiple publications.

### Data analysis

Titles and abstracts of identified publications were screened by trained reviewers (TSB, IJ, SZ, JMdC, LY, AK, and PL). Publications were excluded if they: were duplicates; were not in English; did not report primary data; did not describe a sampling method; did not distinguish between women who are exposed (ie, with circumcised partners) and unexposed (with un-circumcised partners); or did not report a biomedical health outcome.

Publications not excluded by title or abstract screening were passed to full-text screening by trained reviewers (including TSB, IJ, SZ, JMdC, LY, AK, and PL), using the same criteria. Additionally, publications were excluded if they reported an overlapping dataset with a more recent publication or had obvious errors (different results in the abstract and text). Included publications were abstracted by two abstracters into a purpose-built Microsoft Access database (SD; MS Access 2013). When available, e-posters served as data sources. Abstracted data included publication year, study design, inclusion or exclusion criteria, diagnostic methods, sample sizes, and point estimates and uncertainty of associations (appendix 1). Incidence was abstracted preferentially over prevalence, intention-to-treat over other analytical methods, more-adjusted over less-adjusted outcome estimates, and long follow-up periods or late observations in a cohort over short periods or early observations. All quantitative outcome measures were included, such as ratios of incidence rate, prevalence, odds, hazard, and non-ratio and other measures. Outcomes without clinical relevance were not abstracted (eg, individual HPV genotypes). Disagreements were resolved by discussion and, if necessary, through review by the first and senior authors. Results of studies with only qualitative data were planned to be reported for outcomes with no quantitative data (appendix 2).

Estimates of association are presented as comparisons between exposed and unexposed women. Point estimates and CIs in appendix 1 have been inverted when reported in the opposite manner, and CIs not provided were calculated when possible.

After abstraction, datapoints (referring to a single point estimate of association from a specific publication, subgroup, and outcome) were checked for overlap not previously identified on the publication level. When different datapoints reported the same outcome, measure, subgroup, and data collection period in participant groups in the same location with overlapping inclusion criteria, all but one was excluded (excluded points in appendix 1). Priority was given to datapoints that were peer-reviewed, included a superset of participants (rather than a subset), and were more recently published than other datapoints.

**Figure 2:**
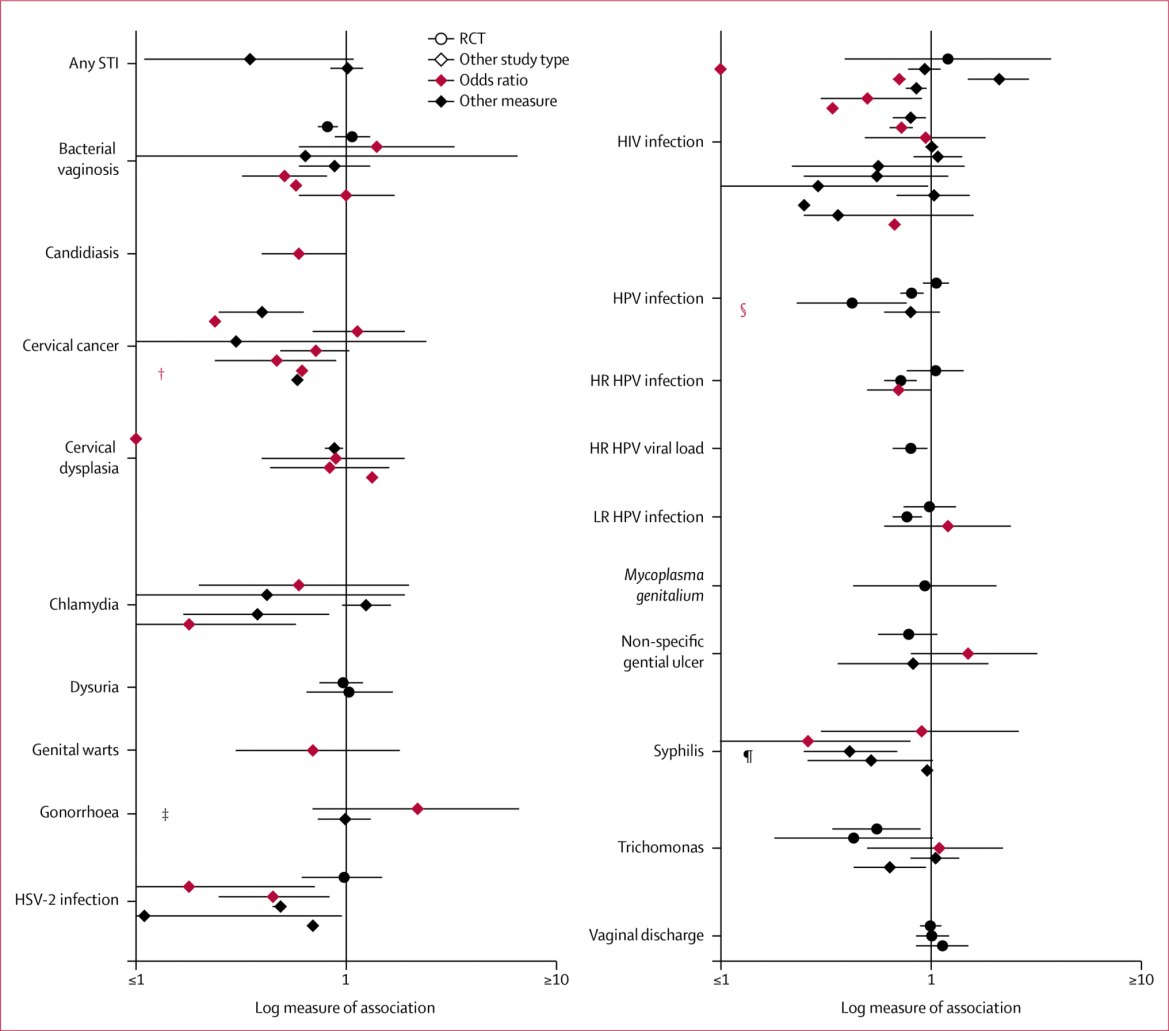
Point estimates of association between male circumcision and women’s health outcomes* STI=sexually transmitted infection. HPV=human papillomavirus. HR=high risk. LR=low risk. HSV-2=herpes simplex virus type 2. RCT=randomised controlled trial. *Datapoints without error bars represent estimates for which confidence intervals were not provided or calculable. †Protective association but no point estimate calculable. ‡No cases in circumcision group.^16^ §All women with uncircumcised partners were positive.^17^ ¶No cases in circumcision group.^16^

Publications listed in appendix 1 were further filtered for display in figure 2. If multiple publications provided different measures of the same outcome in the same study sample (n=4), only the publication with the preferred measure type was displayed (eg, incidence favoured over prevalence). These and non-plottable publications (eg, not providing direction of association) make up the non-plottable publications referenced in the table.^16–70^ Plottable publications constitute the group of independent, interpretable data sources. Only the main result for each outcome was displayed to avoid exaggeration of the number of publications reporting on multiple subgroups. For HIV, four publications reported on subgroups only; the datapoint judged most representative of the target population was selected. Point estimates in figure 2 are highlighted in appendix 1. Point estimates were plotted on a logarithmic scale. Unique symbols (n=4) represent plottable publications with a clear direction of association, which were reported in a form that did not allow point estimate calculation. Their locations reflect only their direction of association.

Quality grading for RCTs used the Grading of Recommendations, Assessment, Development, and Evaluations (GRADE) criteria,^71^ which rank RCTs as providing evidence of high, moderate, low, or very low quality. Quality grading for observational publications used the Newcastle-Ottawa case-control and cohort publication scoring systems, and a Newcastle-Ottawa-derived cross-sectional scale developed elsewhere.^72^ Newcastle-Ottawa systems score quality in three categories: sample selection, comparability between groups, and outcome or exposure assessment;^73^ these categories are combined into a single summary score.

Quality of the overall body of evidence on each outcome was then assessed, with a modified Child Health Epidemiology Research Group^74^ criteria format (table), which included quality of each individual publication’s data on that outcome, magnitude and consistency of associations found across plottable publications, and generalisability of results to the population for which the findings are most relevant (the general female population in sub-Saharan African countries with generalised HIV epidemics). Generalisability was high for outcomes including only studies in these populations, moderate for outcomes including mixed populations, and low for outcomes including only other populations. Consistency of evidence on each outcome was established via a prespecified algorithm incorporating study design and number, and statistical significance (appendix 2). A meta-analysis was not planned.

### Role of the funding source

The funders of this study had roles in study design, data collection, data analysis, data interpretation, and report writing. The corresponding author had full access to all the data in the study and had final responsibility for the decision to submit for publication.

## Results

The flowchart of included publications is shown in figure 1. 112 publications met all inclusion criteria; datapoints from those not included because of population overlap with other publications are listed in appendix 1. Of the remainder, 60 publications had biomedical outcomes, which are summarised in this paper. No outcomes had qualitative data without quantitative data, so qualitative publications were not reviewed (appendix 2).

Populations included groups in Africa, North America, South America, Asia, and Europe, and ages of individuals included ranged from 15 or 18 to 49 or 65 years (appendix 1). Most outcomes included at least some data from African general populations, conferring moderate-to-high generalisability; however, cervical cancer did not. Except in the case of bacterial vaginosis, outcomes had mid-range median-quality scores for observational studies and unclear quality grades for RCTs because they did not meet some of the stringent GRADE criteria (appendix 1). In the remainder of this section, publications not noted to be RCTs were observational, and numbers of datapoints refer to plottable datapoints.

High-consistency evidence was found for five outcomes, which all had protective associations with male circumcision: cervical cancer, cervical dysplasia, HSV-2 infection, chlamydia, and syphilis (figure 2, table). For cervical cancer, nine datapoints were included (none of which were from Africa), conferring low generalisability. All four significant and four of five non-significant datapoints showed a protective association. For cervical dysplasia, five datapoints were included from African and other settings, which conferred moderate generalisability. The two significant and two of three non-significant datapoints showed protective associations. For HSV-2 infection, six data points, one of which was from an RCT, were included from African and other settings, which conferred moderate generalisability. All datapoints (four significant, two non-significant) showed a protective association. For chlamydia, five datapoints were included, which examined participants from African and other settings, conferring moderate generalisability. Both significant and two of the three non-significant datapoints showed a protective association. For syphilis, six data points were included, which examined participants from African and other settings and conferred moderate generalisability. All datapoints showed a protective association, two of which were significant.

Medium-consistency evidence was found for two outcomes, which reported protective associations with HPV infection and low-risk HPV infection. For HPV infection, five datapoints, three of which were RCTs, were included and examined participants from multiple African and European settings, conferring moderate generalisability. Both significant datapoints (two of three RCTs) and two of three non-significant datapoints showed a protective association; the remaining datapoint, an RCT, showed a non-significant harmful association. For low-risk HPV infection, three studies, two of which were RCTs, were included and examined participants from African and European settings conferring moderate generalisability. The significant point, an RCT, showed a protective association, the other RCT showed a non-significant protective association, and the remaining point showed a non-significant harmful association.

Low-consistency evidence was found for seven outcomes because of discrepant values: bacterial vaginosis, gonorrhoea, HIV infection, high-risk HPV infection, non-specific genital ulcers, trichomonas, and vaginal discharge. The six remaining outcomes, with fewer than three studies, were classified as indeterminate consistency: any sexually transmitted infection, candidiasis, dysuria, genital warts, high-risk HPV viral load, and *Mycoplasma genitalium*.

## Discussion

The scale-up of voluntary medical male circumcision in sub-Saharan Africa has been historic, with nearly 15 million circumcisions done between 2007 and 2016.^5^ We aimed to establish which diseases in women had protective associations with male circumcision and to clarify whether scale-up of voluntary medical male circumcision could be relevant to a wide array of women’s health programmes. Our findings show the substantial evidence for the association of male circumcision with decreased risk of several diseases in women.

High-consistency outcomes showed protection associated with circumcision against cervical cancer and dysplasia, chlamydia, HSV-2, and syphilis. Few publications reporting cervical cancer or dysplasia outcomes took place in Africa; however, biological mechanisms underlying protection should be universal. Medium-consistency and low-consistency outcomes are discussed further.

For HPV, publications were from African and European settings. Two of the three RCTs from Rakai, Uganda, reported a protective effect of circumcision, which was significant among long-term partners of HIV-negative males.^9,63^ A third RCT^64^ found a non-significant harmful effect (prevalence ratio 1·06, 95% CI 0·92–1·21) and was unique in that enrolled male partners were HIV positive. Among the other two publications, both found non-significant protective associations: one study^65^ in Spain among women attending routine cervical cancer screenings with two or more lifetime sexual partners, and the other study^17^ in Nigeria among women attending a gynaecological clinic. Publication qualities were mixed—the RCTs and Spanish cross-sectional study^65^ were scored highest. We conclude that the evidence again supports a protective association when male partners are not HIV-infected.

For low-risk HPV, included datapoints were from a subset of the same publications from Uganda and Spain. Of the two RCTs^9,64^ in Rakai that reported low-risk HPV data, one reported a non-significant, minimal-protective effect among partners of HIV-positive men,^64^ and the other reported a significant protective effect among partners of HIV-negative men (95% CI 0·66–0·90).^9^ The Spanish cross-sectional study reported a non-significant, harmful association between partner circumcision and infection. However, the same study reported non-significant protective associations with all-type HPV and high-risk HPV; the association with low-risk HPV might represent chance. Publication qualities were generally high. We conclude that the evidence supports a protective association when male partners are not HIV infected.

For HIV infection—a low-consistency outcome—estimates of association are heavily skewed towards protection. Characteristics of publications not reporting protective associations are informative. The main RCT of circumcision in HIV-positive men showed a non-significant increased risk of HIV acquisition among female partners at 24 months, driven by a significant increased risk among those who resumed sex before their wounds healed. This finding has become a crucial component of pre-circumcision counselling for HIV-positive men.^19^ A cross-sectional study^45^ of pregnant Rwandan women showed a significant harmful association; confounders are not readily apparent, other than the low prevalence of circumcision among male partners (6%), raising the possibility noted by the authors that some were circumcised as treatment for sexually transmitted infections. No obvious confounders exist for the secondary analyses of data from the VOICE^35^ and Hormonal Contraception and the Risk of HIV Acquisition trials.^36^ The remaining 16 trials included publications that showed significant or non-significant protective associations. Excluding the RCT of HIV-positive men, the evidence for protection qualifies as highly consistent. Publications showing both harmful and protective associations had a wide range of qualities.

Bacterial vaginosis, gonorrhoea, high-risk HPV, trichomonas, non-specific genital ulcers, and vaginal discharge were the other low-consistency outcomes. Two patterns underlie this heterogeneity. For gonorrhoea and non-specific genital ulcers, the one data point showing a harmful association was from the same study,^20^ which had a participant pool of members of high-risk populations (recruited from sexually transmitted infection clinics). The datapoints showing harmful associations with trichomonas are from this study^20^ and another study^18^ with patients from sexually transmitted infection clinics. For bacterial vaginosis, of the two datapoints showing a (non-significant) harmful association, the observational point was one of the same two high-risk studies,^20^ and the RCT^19^ enrolled female partners of HIV-positive men. Without this RCT, evidence on bacterial vaginosis would be high consistency for a protective association. For high-risk HPV, the single study^64^ showing a (non-significant) harmful association was also an RCT enrolling partners of HIV-positive men. For vaginal discharge, the same was true for the datapoint showing a harmful association (prevalence ratio 1·13, no CI),^19^ whereas the other two points had prevalence ratio estimates of 0·99^14^ and 1·01.^67^ We conclude that the protective effects of male circumcision for women against many sexually transmitted infections are not evident when male partners are HIV infected. In the case of women at high risk of sexually transmitted infections, the mechanisms that underlie protection in the general population would be expected to operate in the same way, but important confounders might be involved. Alternatively, since these data are derived from the same two studies across all outcomes, their populations might have been unique because of chance.

Any sexually transmitted infection, candidiasis, dysuria, genital warts, high-risk HPV viral load, and *Mycoplasma genitalium* were indeterminate consistency outcomes with fewer than three publications. Research on male circumcision and these outcomes, as well as pregnancy and neonatal outcomes (mediated by associations with transmission of sexually transmitted infection) would be beneficial. Although existing evidence would make further randomisation of men by circumcision status unethical, large observational studies would be valuable. Data on self-reportable outcomes, especially pregnancy outcomes (mediated by sexually transmitted infections), could be collected easily by demographic surveillance studies done for other primary purposes.

We are aware of two publications presenting new data that otherwise qualified for inclusion after the cutoff date for this paper. The first examined community-level HIV incidence in Rakai, Uganda, before and during scale-up of voluntary medical male circumcision and antiretroviral treatment; increasing community-level coverage of voluntary medical male circumcision was associated with a significant reduction in HIV incidence among men and a non-significant reduction in women.^75^ This finding is potentially consistent with models projecting that the reduction in HIV incidence in women due to voluntary medical male circumcision would be delayed relative to those in men.^7,76^ The second publication, which was a baseline analysis of participants in the Partners in Prevention Study,^77^ found that women with circumcised male partners had a lower prevalence of bacterial vaginosis (risk ratio [RR] 0·82, 95% CI 0·72–0·94); including this publication would not have changed the low consistency score.

Included publications rarely presented data permitting determination of whether a direct effect existed; this requires ascertainment of the male partner’s infection status. Available data comes primarily from the Rakai RCT follow-up publications. For HIV, no publication provided significant evidence for a direct effect. Apart from the RCT in HIV-positive men, all publications showed non-significant protective associations,^19,46,47^ and an earlier meta-analysis combined some findings into a significant protective result.^78^ For HSV, results were mixed, with one study supporting^39,40^ and one contradicting^41^ a direct effect. For HPV, a follow-up of the Rakai RCT^63^ found a significant protective effect (adjusted RR 0·42, 95% CI 0·23–0·76) on incident positivity in women for an HPV genotype present in their male partner at baseline, providing the only significant evidence for a direct effect. However, the biological mechanism underlying such an effect is unknown.

Limitations of this paper include, for all outcomes, exclusion of non-English-language publications and near-universal reliance on self-reporting by women of partner circumcision status. We found no information on chancroid, vaginal cancer (mediated by HPV), or pregnancy outcomes; information on some other outcomes was insufficient. Although formal generalisability to the general population of sub-Saharan Africa varied, the mechanistic biological nature of the protection effect makes it plausible that results are widely generalisable. Our decision to include only the main outcome from each publication, to avoid inflation of the apparent weight of publications that analysed multiple subgroups, sometimes resulted in aggregation of different subgroups with divergent point estimates into their single combined estimate. Publications reporting multiple outcomes are more heavily represented in the total evidence than those reporting one. Although objective standards were used for quality assessments, this process has inherent subjective elements. Last, evidence was ranked primarily on the basis of consistency and secondarily on individual study quality. This was a more standardisable approach because of the heterogeneity of study type and number and quality across outcomes; however, this approach is susceptible to publication bias, and for some uses including quantitative estimation of association size by use of pooled data, individual study scores might be more useful.

Male circumcision has relevance not only to HIV prevention but to the context of the broader health needs of women, particularly in sub-Saharan Africa. Male circumcision directly addresses two of the top 20 regional causes of female mortality. HIV/AIDS is the single biggest cause and cervical cancer is the most common cancer in African women;^79,80^ HIV/AIDS is a crucial underlying cause of death from tuberculosis, which is the twelfth biggest direct cause of female mortality. Voluntary medical male circumcision programmes align with the goals of other reproductive and maternal health interventions prioritised by WHO and other organisations—ie, prevention of sexually transmitted infections and HIV, screening and treatment for syphilis,^81^ cervical cancer screening and treatment, and HPV vaccination,^82^ as well as the UN’s Sustainable Development Goals.^83^ Additionally, prevention of syphilis and other sexually transmitted infections in women could prevent associated adverse pregnancy outcomes like stillbirth, low birthweight, preterm birth, and congenital infection.^84^ Such health outcomes should be considered in projections of the cost-effectiveness and impact of voluntary medical male circumcision. The operational intersection between voluntary medical male circumcision and women’s health has also begun to be explored, through demand creation for voluntary medical male circumcision directed at female partners and emphasising its benefits to them,^85^ and PEPFAR’s DREAMS initiative,^86^ which includes voluntary medical male circumcision for male partners among its strategies for HIV prevention in adolescent girls and young women. Possibilities for broader operational synergies between voluntary medical male circumcision and programmes directed at other women’s health outcomes are worth exploring, to take further advantage of the potential of voluntary medical male circumcision to benefit both women and men.

## Supplementary Material

Supplementary appendix 1

Supplementary appendix 2

## Figures and Tables

**Table:** Summary of publications reporting on the association of male circumcision with biomedical health outcomes in women

	Number of publications*	Design(s)	Region(s)	Median quality score † (RCT; observational)	Consistency and direction of evidence	Generalisability to area or population of interest	Generalisability to intervention of interest (eg, traditional male circumcision)
Any STI^16,18^	2	Cross sectional; cohort	Africa, Asia	No RCT; 2/10 to 9/9	Indeterminate	Low	100% self-reported
Bacterial vaginosis^14,16,19–24^	8	RCT; cross sectional	Africa, Asia, USA	Unclear; 2/10 to 4/10	Low	Moderate	50% self-reported
Candidiasis^20^	1	Cross sectional	Africa	No RCT; 6/10	Indeterminate	High	100% self-reported
Cervical cancer^25–33^	9	Case control; cohort	Americas, Asia, Europe, USA	No RCT; 6/9	High protective	Low	50% self-reported or determined on the basis of religion
Cervical dysplasia^17,20,31,32,34^	5	Cross sectional; case control	Africa, Asia, Middle East, USA	No RCT; 5/9	High protective	Moderate	80% self-reported
Chlamydia^16,18,20,35–38^	7 (5 plottable)	Cross sectional; case control; cohort	Africa, Americas, Asia, Europe, USA	No RCT; 6/9	High protective	Moderate	100% self-reported
Dysuria^14,19^	2	RCT	Africa	Unclear; no observational	Indeterminate	High	No self-reporting
Genital warts^20^	1	Cross sectional	Africa	No RCT; 6/10	Indeterminate	High	100% self-reported
Gonorrhoea^16,18,20,35,36^	5 (3 plottable)	Cross sectional; cohort	Africa, Asia	No RCT; 6/10 to 8/9	Low	High	100% self-reported
Herpes simplex virus type 2^33,39–44^	7 (6 plottable)	RCT; cross sectional	Africa, Asia, USA	Unclear; 5/9 to 6/10	High protective	Moderate	60% self-reported
HIV infection^19,20,35,36,45–62^	22 (20 plottable)	RCT; cross sectional; case control; cohort	Africa, Asia, Europe, Middle East	Unclear; 5/9	Low	High	95% self-reported or determined on the basis of location
HPV infection^9,17,63–65^	5	RCT; cross sectional	Africa, Europe	Unclear; 5/10	Medium protective	Moderate	40% self-reported
High-risk HPV infection^9,64,65^	3	RCT; cross sectional	Africa, Europe	Unclear; 5/10	Low	Moderate	33% self-reported
High-risk HPV viral load^66^	1	RCT	Africa	Unclear; no observational	Indeterminate	High	No self-reporting
Low-risk HPV infection^9,64,65^	3	RCT; cross sectional	Africa, Europe	Unclear; 5/10	Medium protective	Moderate	33% self-reported
*Mycoplasma genitalium*^67^	1	RCT	Africa	Unclear; no observational	Indeterminate	High	No self-reporting
Non-specific genital ulcers^20,36,67,68^	4 (3 plottable)	RCT; cross sectional; cohort	Africa	Unclear; 3/10 to 6/10	Low	High	No self-reporting
Syphilis^16,20,33,35,57,69^	6	Cross sectional; cohort	Africa, Asia	No RCT; 6/9	High protective	Moderate	75% self-reported
Trichomonas^14,18–20,36,70^	6 (5 plottable)	RCT; cross sectional; cohort	Africa, Asia	Unclear; 7/9	Low	High	60% self-reported
Vaginal discharge^14,19,67^	3	RCT	Africa	Unclear; no observational	Low	High	No self-reporting

RCT=randomised controlled trial. STI=sexually transmitted infection. HPV=human papillomavirus

* Each row is independent; a publication reporting multiple outcomes is counted once in each outcome row.

† For observational median quality scores, the Newcastle-Ottawa score represents the numerator; the maximum possible score for that study type is the denominator. The two median values are displayed for when an even number of studies were included.

## Research in context

### Evidence before this study

Three randomised controlled trials established that male circumcision provides men who engage in heterosexual sex with partial protection against acquiring HIV and some sexually transmitted infections. In 2007, WHO recommended that 14 countries with high prevalence of HIV and low prevalence of circumcision should scale-up male circumcision as an additional HIV prevention strategy; nearly 15 million men and boys were circumcised in HIV prevention programmes up to 2016. Observational studies and follow-up research at original clinical trial sites have shown that male circumcision also decreases the risk of HIV, some sexually transmitted infections, and other adverse health outcomes in female partners of circumcised men. Given the magnitude of the male circumcision programme for HIV prevention in sub-Saharan Africa, the potential impact on women’s health is substantial. However, estimates of association vary among studies and the broad effect across numerous women’s health outcomes have not been characterised.

### Added value of this study

We found that female partners of circumcised men are less likely to have various adverse health outcomes, including multiple sexually transmitted infections. Evidence that male circumcision is associated with decreased risk of cervical cancer, cervical dysplasia, herpes simplex virus type 2 infection, chlamydia, and syphilis in women was highly consistent. Evidence that male circumcision is associated with decreased risk of human papillomavirus (HPV) infection and low-risk HPV infection in women was of medium consistency. The weight of evidence also supports the protective association between male circumcision and HIV in women.

### Implications of the available evidence

The scale-up of the male circumcision programme has potential benefits for women’s health. Strengthening of programmatic linkages and synergies between male circumcision and women’s health programmes, including cervical cancer prevention, could be maximised in settings in which prevalence of sexually transmitted infections and cervical cancer is high. Policy makers, programme implementers, and researchers could explore these linkages further to ensure that the benefits of male circumcision for women’s health are fully optimised.

## *Panel:* MEDLINE search strategy

“Circumcision”, “Male/” OR “vmmc.ti,ab.” OR (“circumcis*” ADJ10 [“male*” OR “men” OR “man” OR “boy*”]).ti,ab. OR ([“foreskin*” OR “prepuce*”] ADJ5 [“penis” OR “penile” OR “male” OR “man” OR “men” OR “boy*”]).ti,ab. AND “Exp Women’s Health/” OR “Women/” OR (“women” OR “woman” OR “female*” OR “partner*” OR “wife*” OR “wives” OR “sex worker*” OR “prostitute*” OR “girl*” OR “mother*” OR “daughter*”).ti,ab.
