# Supplementary material for: Association between male circumcision and women’s biomedical health outcomes: a systematic review
Source: Lancet Glob Health. Author manuscript; Available in PMC 2017 Dec 13. (PMC5728090; doi:10.1016/S2214-109X(17)30369-8)
Supplement: Supplementary appendix 2 [file NIHMS922992-supplement-Supplementary_appendix_1.pdf]

THE LANCET
Global Health

Supplementary appendix

This appendix formed part of the original submission and has been peer reviewed. We post it as supplied by the authors.

Supplement to: Grund JM, Bryant TS, Jackson I, et al. Association between male circumcision and women's biomedical health outcomes: a systematic review. *Lancet Glob Health* 2017; **5:** e1113–22.

Qualitative publications passing screening, excluded because quantitative data available on the same outcome

Abbott SA, Haberland NA, Mulenga DM, Hewett PC. Female Sex Workers, Male Circumcision and HIV: A Qualitative Study of Their Understanding, Experience, and HIV Risk in Zambia. *PLoS ONE* 2013*;* 8(1).

Arnott J, Koehler J. Medical Male Circumcision for HIV Prevention: Are Women Ready? 2010 report for AVAC and WHiPT. Available online at https://www.malecircumcision.org/resource/medical-male-circumcision-hiv-prevention-are-women-ready.

Bailey R., Muga R., Poulussen R., Abicht, H. The acceptability of male circumcision to reduce HIV infections in Nyanza Province, Kenya. *AIDS Care* 2002*;* 14(1), 27-40.

Castro JG, Jones D, Barradas I., Lopez M., Weiss S. Attitudes towards neonatal male circumcision among Hispanic men and women in Miami, Florida. *Int J Inf Dis* 2010*;* 14, e77.

Castro, J. G., Jones, D. L., Lopez, M., Barradas, I., & Weiss, S. M. Making the case for circumcision as a public health strategy: opening the dialogue. *AIDS Patient Care and STD* 2010; 24(6), 367-372.

Castro-Vazquez, G. Paediatric male circumcision and penile hygiene: a Japanese mothers' view. *Anthropology & Medicine* 2013, 20(3), 299-310.

Chikutsa A., Maharaj P. Social representations of male circumcision as prophylaxis against HIV/AIDS in Zimbabwe. *BMC Public Health* 2015; (15): 603.

Goicochea P, Casapia M, Morales M. Understanding the cultural meaning of the penis prepuce in the Amazon region of Peru: a qualitative approach. Washington DC. AIDS 2012 Abstract WEPE252.

Greely P, Maharaj P, Letsoalo T, Miti, A. Traditional male circumcision for reducing the risk of HIV infection: perspectives of young people in South Africa. *Culture, Health & Sexuality* 2013; 15(2), 148-159.

Haberland NA, Kelly CA, Mulenga DM, Mensch BS, Hewett PC. Women’s Perceptions and Misperceptions of

Male Circumcision: A Mixed Methods Study in Zambia. *PLoS ONE* 2016; 11(3): e0149517. doi:10.1371/journal.pone.0149517

Hoffman JR, Arendse KD, Larbi C, Johnson N, Vivian LM. Perceptions and knowledge of voluntary medical male circumcision for HIV prevention in traditionally non-circumcising communities in South Africa. *Glob Public Health* 2015;10 (5-6):692-707. doi: 10.1080/17441692.2015.1014825. Epub 2015 Mar 2.

Jarrett, P., Kliner, M., & Walley, J. (2014). Early infant male circumcision for human immunodeficiency virus prevention: knowledge and attitudes of women attending a rural hospital in Swaziland, Southern Africa. *SAHARA J: Journal of Social Aspects of HIV/AIDS Research Alliance* 2014; 11(1), 61-66.

Kaufman M, Dam K, Hatzold K, et al. Adolescent VMMC Technical Advisory Group. Adolescent girls' support of male peers and sexual partners receiving voluntary medical male circumcision services: implications for demand creation. Poster presented at International AIDS Society Conference 2016, July 18-22, 2016, Durban, South Africa. Available online at http://programme.aids2016.org/Abstract/Abstract/5516.

Lanham M., L'Engle K, Loolpapit M., Oguma, IO. Women's roles in voluntary medical male circumcision in Nyanza Province, Kenya. *PLoS ONE* 2012; *7*(9), e44825.

Layer EH, Beckham SW, Mgeni, L, Shembilu C, Momburi RB, Kennedy CE. "After my husband's circumcision, I know that I am safe from diseases": women's attitudes and risk perceptions towards male circumcision in Iringa, Tanzania. *PLoS ONE* 2013; *8*(8), e74391.

Layer EH, Beckham SW, Momburi, RB, Kennedy CE. Understanding the partial protection of male circumcision for HIV prevention among women in Iringa Region, Tanzania: an ethnomedical model. *AIDS Care* 2013*; 25*(8), 1045-1050.

Lukobo M., Bailey, R. Acceptability of male circumcision for prevention of HIV infection in Zambia. *AIDS Care* 2007*; 19*(4), 471-477.

Mantell JE, Smit JA., Saffitz, JL, Milford C, Mosery N, Mabude,Z, Stein ZA. Medical male circumcision and HIV risk: perceptions of women in a higher learning institution in KwaZulu-Natal, South Africa. *Sexual Health* 2013; *10*(2), 112-118.

Ngalande RC, Levy J, Kapondo CP, Bailey RC. Acceptability of male circumcision for prevention of HIV infection in Malawi. *AIDS & Behavior* 2006, *10*(4), 377-385.

Rennie S, Perry B, Corneli A, Chilungo A, Umar E. Perceptions of voluntary medical male circumcision among circumcising and non-circumcising communities in Malawi. *Glob Public Health.* 2015; 10(0): 679–691. doi:10.1080/17441692.2015.1004737.

Riess, TH, Achieng MM, Bailey RC. Women's Beliefs about Male Circumcision, HIV Prevention, and Sexual Behaviors in Kisumu, Kenya. *PLoS ONE* 2014; *9*(5), e97748.

Riess TH. Exploring the Relationship between Male Circumcision and Sexual Risk Compensation in Kisumu, Kenya. Thesis for the degree of Doctor of Philosophy in Public Health Sciences in the Graduate College of the University of Illinois at Chicago, 2014.

Scott B, Weiss H., Viljoen, J. The acceptability of male circumcision as an HIV intervention among a rural Zulu population, KwaZulu-Natal, South Africa. *AIDS Care* 2005; *17*(3), 304-313.

Taljaard D, Rain-Tljaard R, Nhlapo C, Auvert B. Female parents and partners to an HIV male circumcision intervention. Mexico City - AIDS 2008. Abstract no. MOPE0546.

Tarimo, E. A., Francis, J. M., Kakoko, D., Munseri, P., Bakari, M., & Sandstrom, E. (2012). The perceptions on male circumcision as a preventive measure against HIV infection and considerations in scaling up of the services: a qualitative study among police officers in Dar es Salaam, Tanzania. . *BMC Public Health*, *12*, 529.

Waters E, Mugisa B, Li M, Linyama D. Acceptability and uptake of neonatal male circumcision in Lusaka, Zambia. Poster *TULBPE054* presented at IAS 2011, Rome.

Waters E, Stringer E, Mugisa B, Temba S, Bowa K., Linyama, D. Acceptability of neonatal male circumcision in Lusaka, Zambia. *AIDS Care* 2012; *24*(1), 12-19. doi:10.1080/09540121.2011.587508

Detailed Methods Supplement:

Determining consistency: Consistency of evidence for each outcome was determined on the following basis, using the data points included in Figure 2. Abstracted outcomes were highly consistent if they met one of three criteria: if data points from two or more RCTs were present, all agreed on the significance of effect, and the direction if significant. Otherwise, if there were data points from ≥4 publications, then ≥75% of point estimates agreed on direction of effect, including 2/3 of any RCTs; or all point estimates agree on direction of effect if data points from 3 publications were present. Among outcomes for which evidence was not highly consistent, those with data points from ≥3 publications could be considered for medium consistency. In order to have medium consistency evidence, outcomes must have had at least 67% of point estimates agree on the direction of effect, including the 2 with the highest quality scores (as a percentage of total potential points), the one with the largest sample size, and any RCTs (or 67% of RCTs if there are ≥3). Outcomes for which there were three or more data points but they did not fulfill these criteria were considered to have low-consistency evidence. Those for which there were fewer than three data points were considered to have indeterminate-consistency evidence.

Designation of study design type: In most cases this was the study design reported in the publication. However, studies involving secondary analyses of participant characteristics from RCTs which randomized participants on another variable than circumcision were considered cohort or cross-sectional studies with respect to circumcision, rather than RCTs. Similarly, follow-up publications to RCTs of circumcision which collected pre-post data in the circumcision group rather than making comparisons to the control group were considered pre-post studies.

Identification of duplicate data points: The goal of the supplementary tables was to make available results on all groups and subgroups for which they were available. For that reason, data points which were otherwise duplicates were not considered duplicated if they contained reports on different subgroups, or a main group and its subgroups, and therefore different results in the tables should not be considered to all necessarily reflect independent data.

Outcome data points were considered duplicates of each other if they were the same outcome or sub-outcome, reported in the same group or subgroup; and the publications in which they were found derived data was from the same geographic location, and had inclusion criteria such that the same participants could reasonably have been included in both. In most cases this resulted from the publication of multiple publications from the same study or series of studies.

Once identified, duplicates were then potentially excluded from the outcome-specific tabs in Appendix 1, based on the below criteria. Where nonrandomized longitudinal studies reported on associations of both baseline and incident outcomes with male circumcision, the point estimates for incident outcomes were included in the figure.

Hierarchy of duplicate data points: If duplicate data points were found, the following rules were used to determine which was retained in the main supplementary table; the others were moved to the supplementary table of excluded outcomes (supplementary table S3). If the data points reported different data collection time periods, then if those time periods were nested (e.g. 2000-2010 and 2005-2008), the source that captured the widest time range was retained. Peer-reviewed manuscripts were retained over non-peer-reviewed results (abstract, dissertation, white paper). Where both of a set of duplicate data points were peer-reviewed, the one with the later publication date was retained.

Figure 2: The goal of the figure was to intuitively display the distribution of the evidence for each outcome. For that purpose it was necessary that each set of data appear only once. For each outcome, only the data point for

the main participant group (not subgroups) from each publication was chosen. When a publication provided no main group estimate, we picked the group most resembling the general population (e.g. HIV- women) rather than inflating the apparent weight of that publication by showing multiple subgroups. This occurred in four cases, all reporting on the HIV outcome: Wawer et al,[19] where the subgroup with delayed post-MC resumptions of sex was chosen over the subgroup with early resumption; Babalola et al[50] where countries with high prevalence of male circumcision rather than those with medium prevalence were chosen for comparison to those with low prevelance; Cuadros et al[58] where the more recent dataset was chosen over the older one for comparison of areas with low MC coverage to those with high MC coverage; and Chemtob et al[60] where the subgroup with the most recent data was chosen, comparing Israel to the Netherlands rather than to France.

If no main group result was provided and no subgroup was more representative of the general population, but subgroup results were consistent in direction, the less extreme result is displayed. If in this situation no subgroup was more representative of the general population, and the direction of results was consistent between subgroups, the least extreme result was displayed. Conversely, if subgroup results were inconsistent in direction (N=2), the publication was excluded from the graph as being impossible to capture in one data point. In situations where a publication reported results over two or more non-overlapping time periods, we chose to represent the most recent time period in the figure to favor reflecting the effects of VMMC programs rather than traditional circumcision practices. Where a publication reported both a prevalence ratio and unadjusted incidences for the main group (N=1), we reported the incidence rather than prevalence ratio. If direction of effect was not reported (N=2), the result could not be shown.

Confidence intervals not provided in publications were calculated where possible. Because adjusted estimates were preferentially abstracted, confidence intervals were not always calculable in these cases even where numbers of events and participants were provided.

Quality grading: For cohort studies, the level of loss to follow-up deemed ‘acceptable’ was 20% or lower. All domains were considered “key” in assigning final quality levels to RCTs using the GRADE criteria.

Other: For the one included abstract from which the data was subsequently published in a manuscript with refined analyses[70] (Pintye et al, *Trichomonas vaginalis*), numerical data in the table and figure represent the updated calculations in the manuscript. (Pintye J, Drake AL, Unger JA, et al. Male Partner Circumcision associated with lower *Trichomonas vaginalis* incidence among pregnant and postpartum Kenyan women: a prospective cohort study. Sex Transm Infect 2017;93:137–143.)
